# A photorealistic synthetic stereo dataset with ground-truth depth and 6-DoF poses

**DOI:** 10.1016/j.dib.2026.113222

**Published:** 2026-09-03

**Authors:** Yan Medeiros Morona, Tiago Loureiro Figaro da Costa Pinto, Daniel Juchem Regner

**Affiliations:** Federal University of Santa Catarina (UFSC), Department of Mechanical Engineering, Florianopolis, SC, Brazil

**Keywords:** Stereo vision, Synthetic dataset, Unreal Engine, AirSim, Depth ground truth, 6-DoF trajectory, SLAM benchmarking

## Abstract

This data article presents a synthetic stereo vision dataset composed of synchronized RGB stereo images, ground-truth depth maps, and precise 6-DoF ground-truth poses generated using Unreal Engine 4 integrated with the AirSim simulation framework. Four indoor virtual scenes resembling industrial and enterprise-like environments were created, including structural elements such as pipes, pillars, walls, furniture, and occlusions. Multiple acquisition configurations were executed as closed-loop drone trajectories, resulting in a total of 36,838 stereo image pairs. The dataset includes multiple trajectory smoothness conditions, stereo baselines, and camera convergence configurations. All RGB images have a fixed resolution of 640 × 480 pixels and are provided alongside pixel-aligned depth maps and time-stamped ground-truth poses. The dataset also includes association files linking stereo images, depth maps, and poses, following formats commonly used in visual SLAM benchmarks. The acquisition is scripted and free of stochastic components, enabling benchmarking and development of stereo depth estimation, visual odometry, and SLAM algorithms under controlled and repeatable indoor conditions.

Specifications Table

**Table:** 

Subject	Engineering & Materials science
Specific subject area	Stereo vision and depth estimation from synthetic images*.*
Related research article	None.
Type of data	Stereo RGB images; depth maps; pose files; association files; camera calibration files; Folder-structured dataset. Formats: PNG (RGB), NPY (depth), txt (poses), JSON (camera parameters).
Data collection	Data were collected in Unreal Engine 4 using Microsoft AirSim. A fixed stereo camera rig was mounted on a virtual agent following scripted trajectories inside four custom-designed indoor scenes. AirSim provided synchronized RGB images, metric depth maps from the depth camera API, and precise 6-DoF poses of the vehicle platform. All captures were automated using Python scripts via the AirSim API*.*
Data source location	Federal University of Santa Catarina (UFSC), Florianopolis, SC, Brazil.
Data accessibility	Repository name: Hugging Face Datasets Data identification number: 10.57967/hf/8959 Direct URL to data: https://huggingface.co/datasets/yanmorona/UE4-Stereo License: Creative Commons Attribution 4.0 International (CC BY 4.0) Total size: 81.8 GB

## 1 Value of the Data

• These data provide synchronized stereo RGB images with perfectly aligned ground-truth depth maps and 6-DoF poses, enabling accurate benchmarking of stereo vision algorithms, visual odometry, and SLAM methods.
• Multiple acquisition configurations, including variations in trajectory direction, drone displacement, stereo baseline, camera convergence, trajectory smoothness, and temporal density, allow controlled benchmarking of geometric conditions, temporal consistency, and trajectory robustness in stereo vision and SLAM pipelines.
• Other researchers can reuse the scenes and routes to evaluate domain adaptation, synthetic-to-real transfer, robustness to occlusions, and generalization of transformer-based stereo architectures.
• The structure of the dataset is compatible with widely used stereo and SLAM frameworks, including ORB-SLAM3 [1].
• The dataset fills a gap between highly abstract indoor synthetic datasets and real-world industrial scenarios, providing a controlled environment with realistic textures, lighting, and clutter.

## 2 Background

Stereo vision and visual SLAM research often rely on datasets that either focus on outdoor scenarios or provide limited control over acquisition parameters. Synthetic environments generated with modern game engines enable precise control of geometry, motion, and sensor configuration while providing accurate ground truth. Unreal Engine 4 combined with the Microsoft AirSim simulation framework enables deterministic execution of camera trajectories and direct access to synchronized RGB images, depth data, and ground-truth poses [2,3]. Recent synthetic datasets, such as MESA [4], further demonstrate the relevance of simulation-based data for visual SLAM evaluation in controlled environments. The dataset presented here follows this synthetic-data paradigm while focusing on stereo image acquisition with dense ground-truth depth, multiple camera configurations, and closed-loop trajectories in indoor environments.

Existing benchmarks either provide real imagery with sparse or indirect depth references, as in KITTI [5] and EuRoC [6], or synthetic imagery with dense and exact ground truth, as in TartanAir [7], Replica [8] and MESA [4]. In all of them the stereo configuration is fixed by construction, so the sensitivity of an algorithm to the acquisition geometry itself cannot be isolated. The dataset presented here complements these resources by holding the environment and the route constant while varying stereo baseline, camera convergence, loop direction and temporal sampling, which allows the effect of each acquisition parameter to be assessed independently of scene content. Table 1 summarises this comparison.

**Table 1:** Positioning of the UE4-Stereo dataset with respect to widely used stereo and SLAM benchmarks.

Dataset	Environment	Depth GT	Stereo geometry	Pose GT
KITTI [5]	Outdoor, driving	Sparse LiDAR	Fixed	GPS/INS
EuRoC MAV [6]	Indoor, MAV	Not provided	Fixed	Motion capture / laser
TartanAir [7]	Synthetic, varied	Dense, exact	Fixed	Exact
Replica [8]	Synthetic, indoor	Dense, exact	Not stereo-specific	Exact
MESA [4]	Synthetic, indoor	Dense, exact	Fixed	Exact
UE4-Stereo	Synthetic, indoor	Dense, exact	Variable (24/32 cm; parallel/convergent)	Exact

## 3 Data Description

The dataset comprises four indoor scenes, 32 acquisition sequences, 36,838 synchronized stereo pairs, corresponding depth arrays, and full 6-DoF ground-truth trajectories, covering variations in loop direction, baseline, camera convergence, and lateral displacement.

The complete dataset occupies approximately 81.8 GB and is distributed under the Creative Commons Attribution 4.0 International licence. Individual scenes and sequences can be downloaded separately, so users interested in a subset of the acquisition configurations do not need to retrieve the full archive.

All RGB images have a fixed resolution of 640 × 480 pixels, three color channels (RGB), and are stored in PNG format. Ground-truth depth maps are stored as 32-bit floating-point NPY files aligned with the left camera. Camera poses are provided in text format, and calibration parameters are stored in JSON files.

All sequences are closed-loop trajectories, where the drone takes off, traverses the loop, and lands at the same initial location. Sequences are acquired under controlled variations in: (i) loop direction, (ii) drone displacement, (iii) stereo baseline, and (iv) camera convergence.

**Fig. 1:**
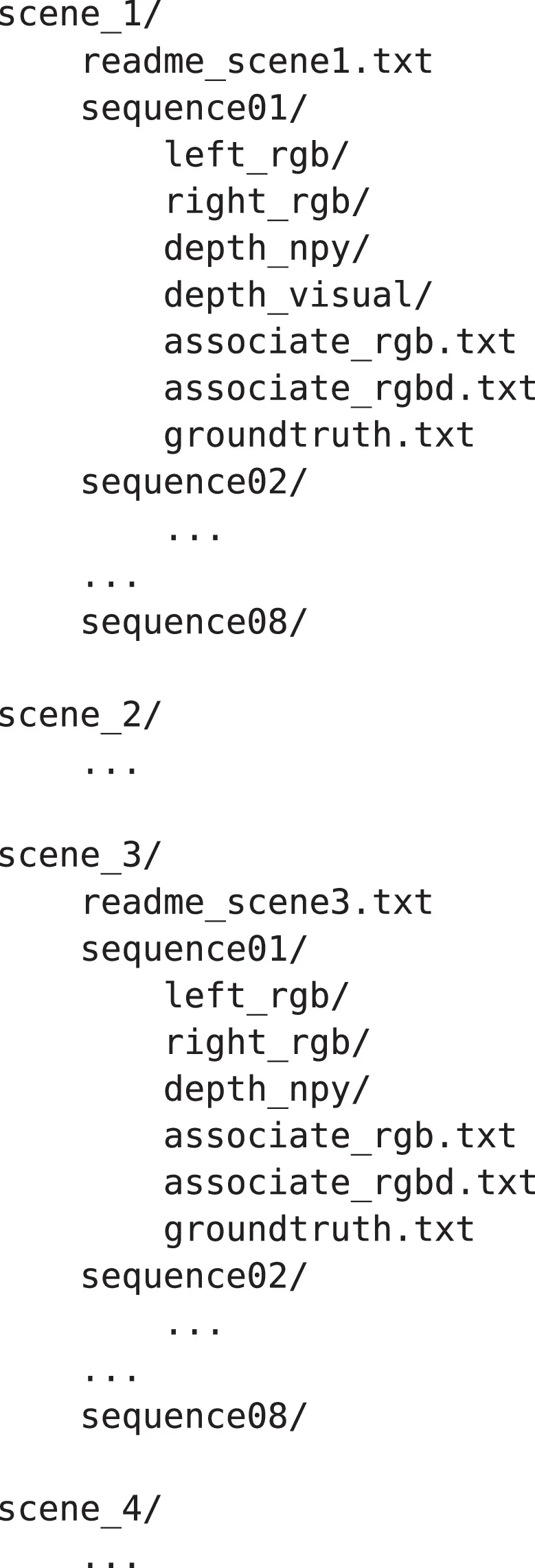
Folder hierarchy and file organization of the UE4-Stereo dataset.

Each scene has a base folder containing a readme and eight sequence folders. The dataset follows the folder structure shown in Fig. 1:

Data formats and contents (per sequence)

**Fig. 2:**
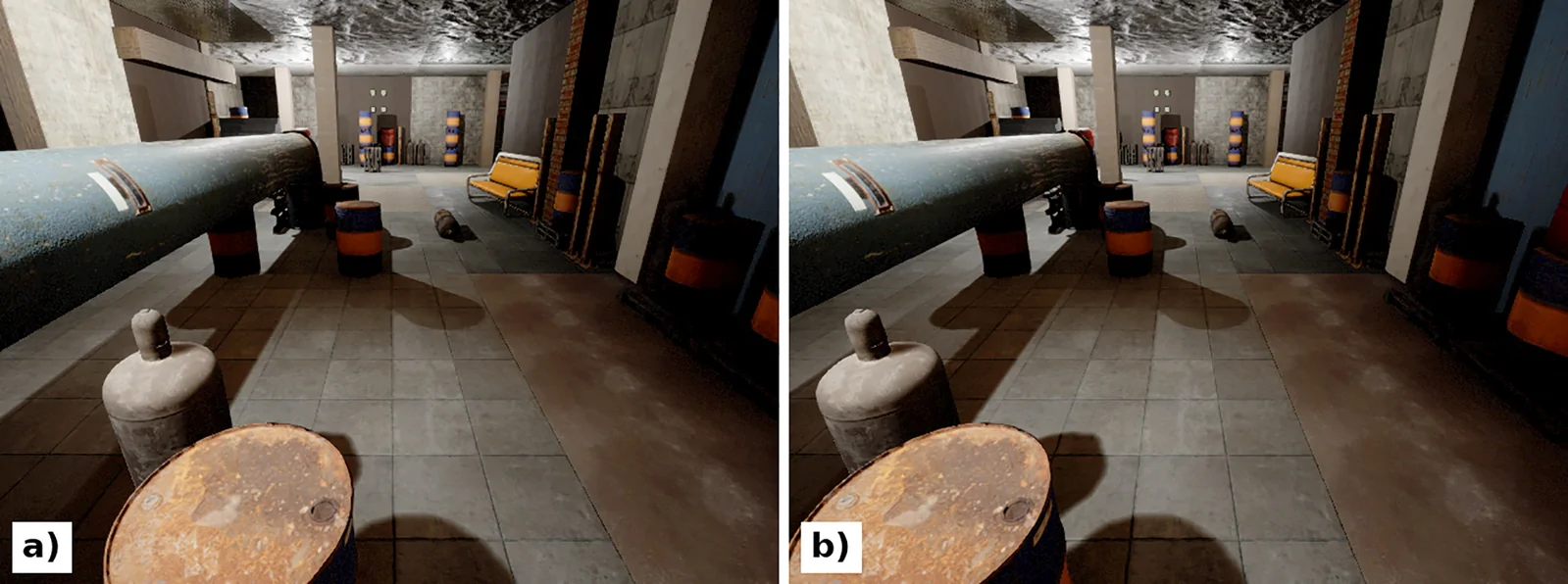
Sample stereo RGB image pairs extracted from scene_1 a) left camera and b) right camera.
2) right_rgb/ Right camera RGB images in PNG format, 640 × 480 pixels, 3 channels (RGB). Each file corresponds one-to-one with a left image via the stereo association file (Fig. 2b).
3) depth_npy/ Ground-truth depth is provided as NumPy arrays stored in .npy format. Each file contains a dense depth map with dimensions 640×480, stored as 32-bit floating-point values, encoding metric depth in meters. Each depth array is pixel-wise aligned with the corresponding left RGB image.
  Depth is acquired with the AirSim DepthPlanar image type from the left camera. Each value represents the orthogonal distance from the camera plane to the observed surface along the optical axis, rather than the radial distance to the optical centre, and is therefore directly comparable with disparity through the relation Z = fx B / d, with fx and B given in Table 3. Pixels for which the simulator returns a non-finite value are set to zero and should be treated as invalid.
4) depth_visual/ For each depth array in Scenes 1 and 2, a corresponding visualization image is provided in PNG format (see Fig. 3). These images have the same spatial resolution (640×480) and store normalized depth values as 8-bit grayscale intensities. The visualization files are intended solely for qualitative inspection and illustration purposes and do not preserve the absolute metric depth values encoded in the .npy files. Visualization images are not provided for Scenes 3 and 4; the corresponding metric depth arrays are available for all sequences of all four scenes.

1) left_rgb/ Left camera RGB images in PNG format, 640 × 480 pixels, 3 channels (RGB). Filenames follow a time-based naming convention produced during simulation (Fig. 2a).

**Fig. 3:**
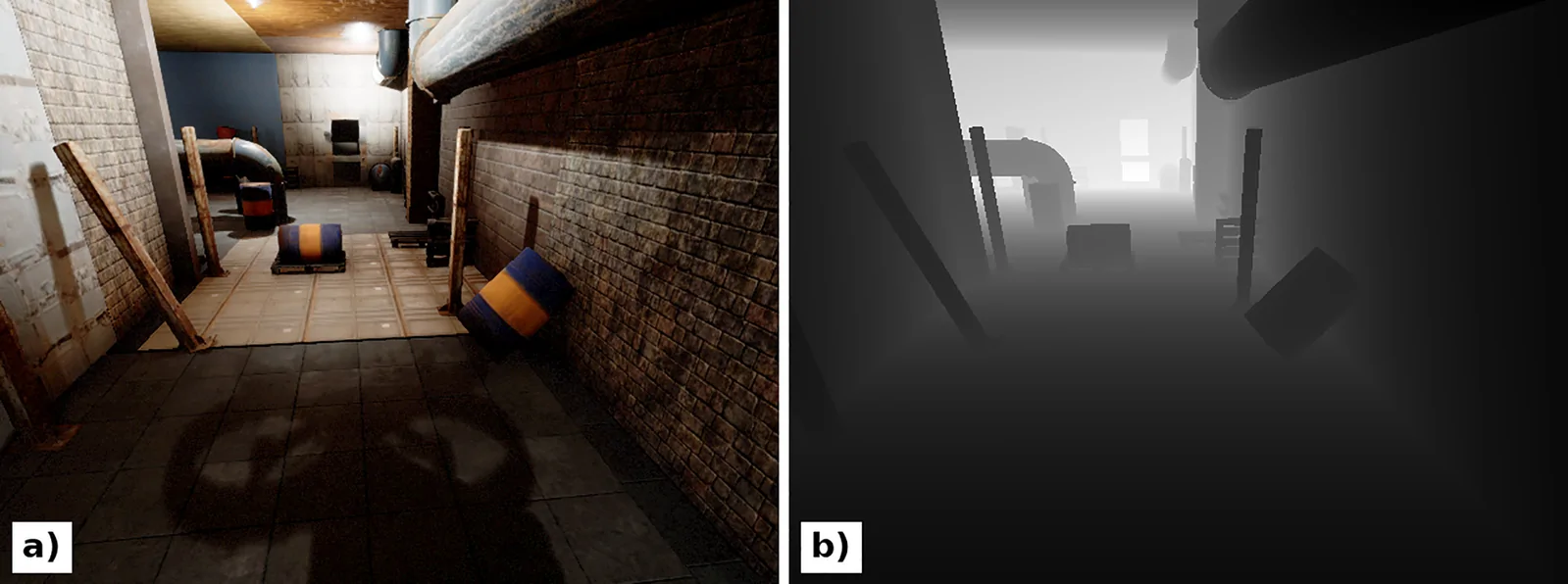
Example of an RGB image (a) and its corresponding reference metric depth map (b).
5) associate_rgb.txt Stereo association file listing synchronized left and right RGB images. Each line follows: timestamp_left path_left timestamp_right path_right.
6) associate_rgbd.txt RGB–depth association file linking each left RGB image to its depth array. Each line follows: timestamp_rgb path_rgb_left timestamp_depth path_depth.
7) groundtruth.txt The dataset provides full 6-DoF ground-truth poses generated in simulation. Each pose is represented by a timestamped rigid-body transformation following the standard TUM RGB-D format [9]:
  # timestamp tx ty tz qx qy qz qw.

where (tx, ty, tz) is the position and (qx, qy, qz, qw) is the orientation quaternion. The recorded transformation describes the pose of the vehicle body frame, to which the stereo rig is rigidly attached. The optical frames of the left and right cameras are obtained by composing this pose with the fixed extrinsic transform reported in Table 3, which specifies the translation and rotation of each camera with respect to the body frame. A single timestamp is shared by the left image, the right image, the depth array and the pose of the same frame, so no temporal interpolation is required to align the modalities.

**Fig. 4:**
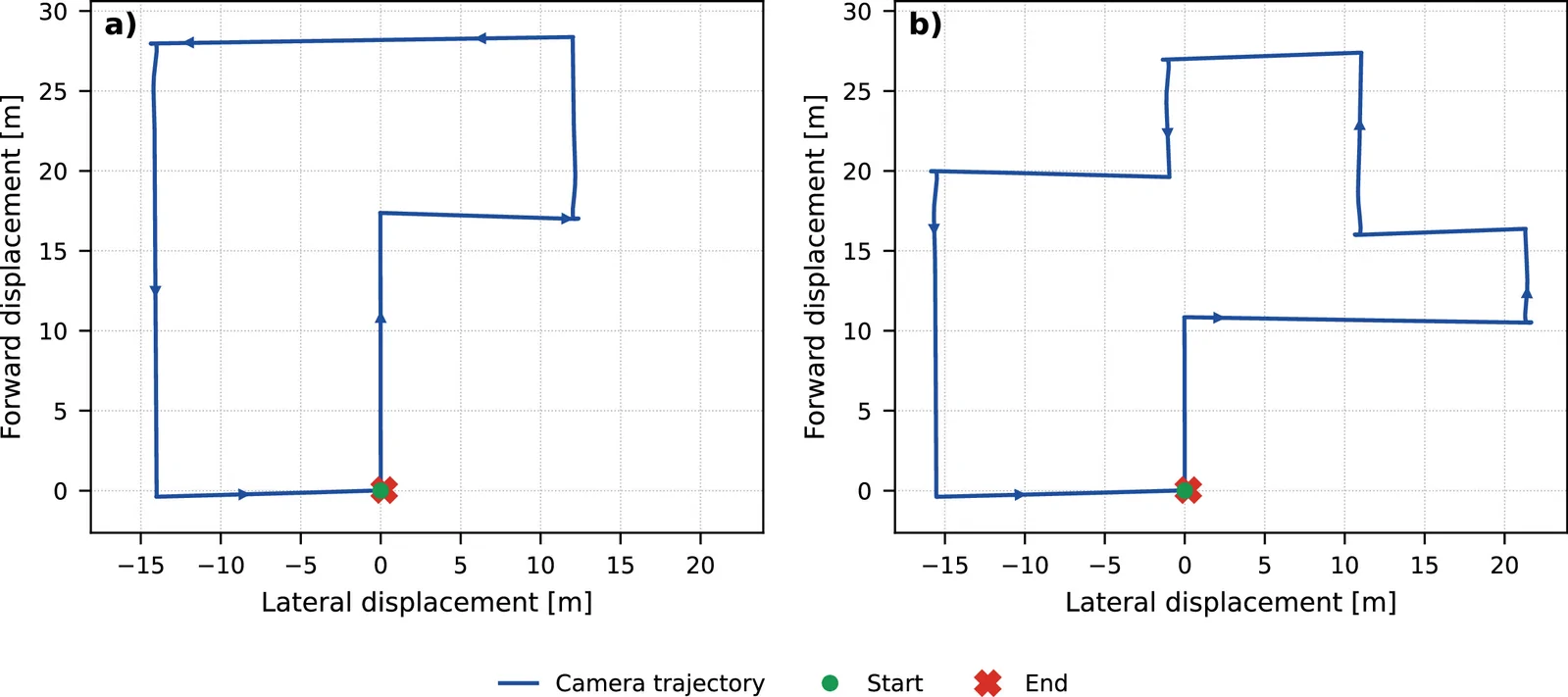
Example of top-down camera trajectory visualization projected onto the horizontal plane (XY) for a) Scene 1 and b) Scene 2.

Ground-truth poses are expressed in the AirSim world frame, a right-handed North-East-Down (NED) coordinate system in metres whose origin coincides with the take-off point of each sequence. This frame differs from the native Unreal Engine world frame, which is left-handed and expressed in centimetres. For visualization purposes, trajectories are commonly represented using a top-down projection onto the horizontal plane (XY), which facilitates inspection of the closed-loop motion patterns (see Fig. 4). This projection is intended solely for illustrative visualization and does not modify or reinterpret the underlying 6-DoF pose data provided in the dataset.

Example Python scripts demonstrating how to load and visualize the stereo images, the metric depth arrays and the ground-truth trajectories are provided in the repository, together with the acquisition scripts used to generate the dataset.

### 3.1 Scene configuration summary

The eight sequences in each scene are defined as follows.

In every scene, four sequences follow a centred route and four follow a displaced route. Displaced routes are laterally offset by approximately 1 m with respect to the centred ones and, in some scenes, are additionally flown at a slightly different altitude. All trajectories are executed between approximately 2 m and 2.5 m above the take-off point. The realized altitude profile of each individual sequence is contained in the corresponding ground-truth trajectory.

Scene 1 (8 sequences, total 4407 stereo pairs). Representative stereo pairs and depth maps for each of the eight sequences of Scene 1 are shown in Fig. 5, Fig. 6, Fig. 7, Fig. 8, Fig. 9, Fig. 10, Fig. 11, Fig. 12.

**Fig. 5:**
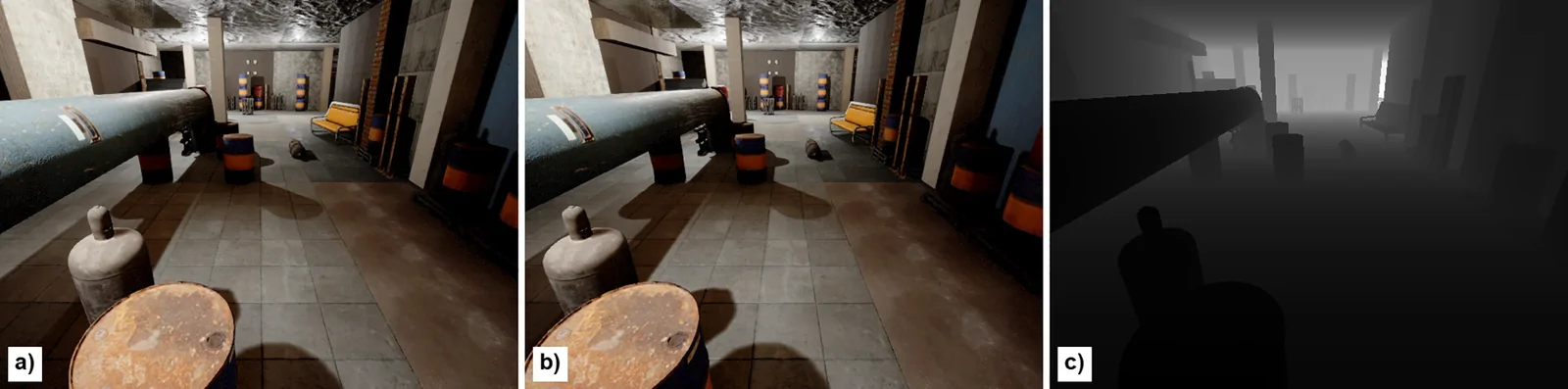
sequence01: direction 1, centered, baseline 24 cm, parallel cameras (532).

**Fig. 6:**
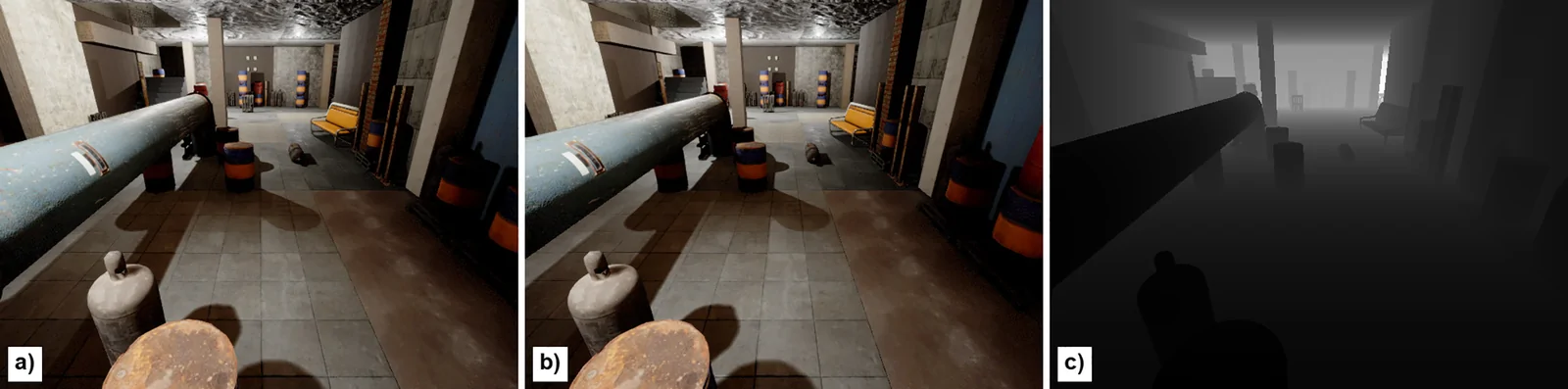
sequence02: direction 1, displaced, baseline 24 cm, parallel (522).

**Fig. 7:**
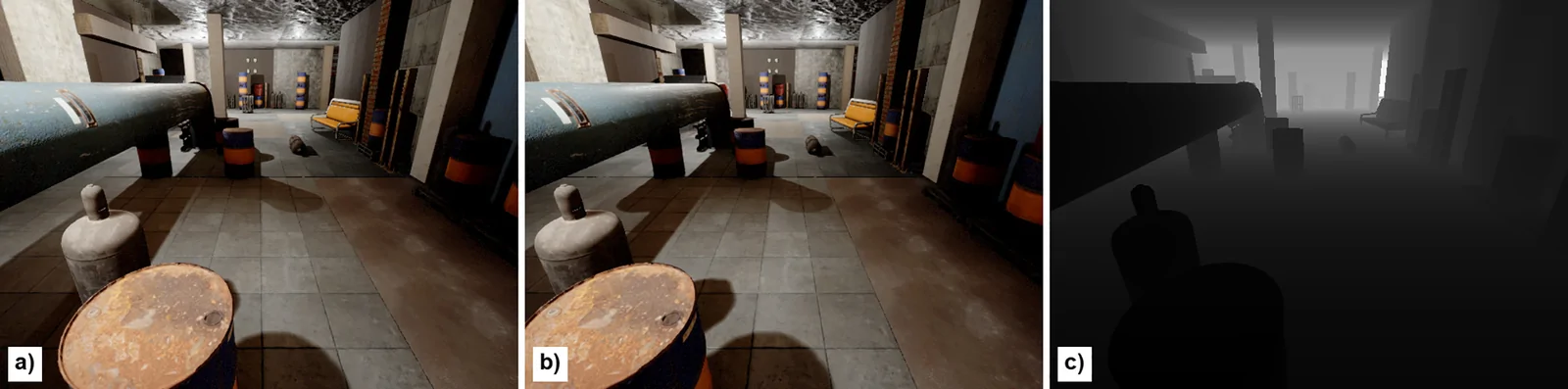
sequence03: direction 2, centered, baseline 24 cm, parallel (548).

**Fig. 8:**
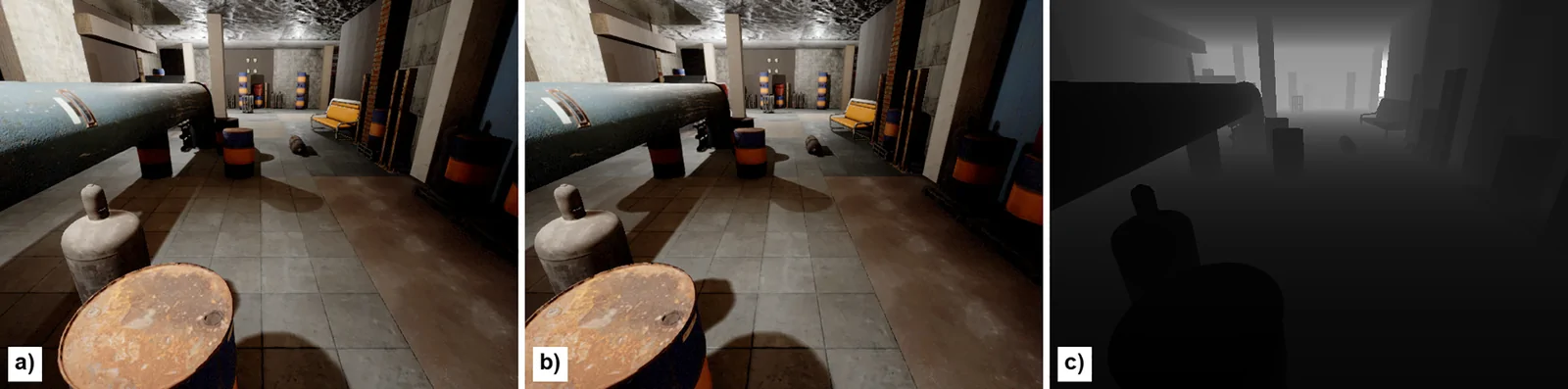
sequence04: direction 2, displaced, baseline 24 cm, parallel (581).

**Fig. 9:**
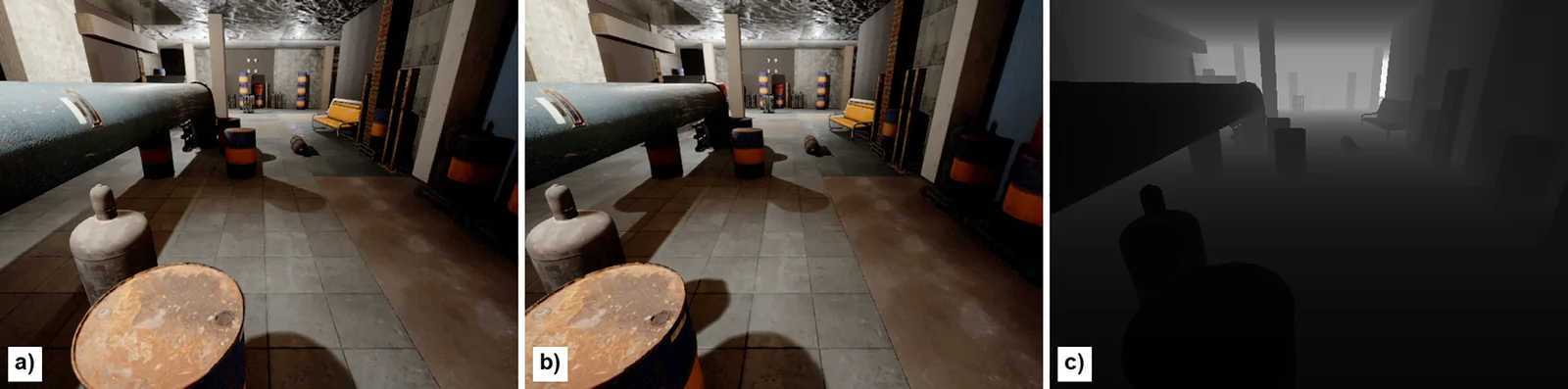
sequence05: direction 1, centred, baseline 32 cm, parallel (560).

**Fig. 10:**
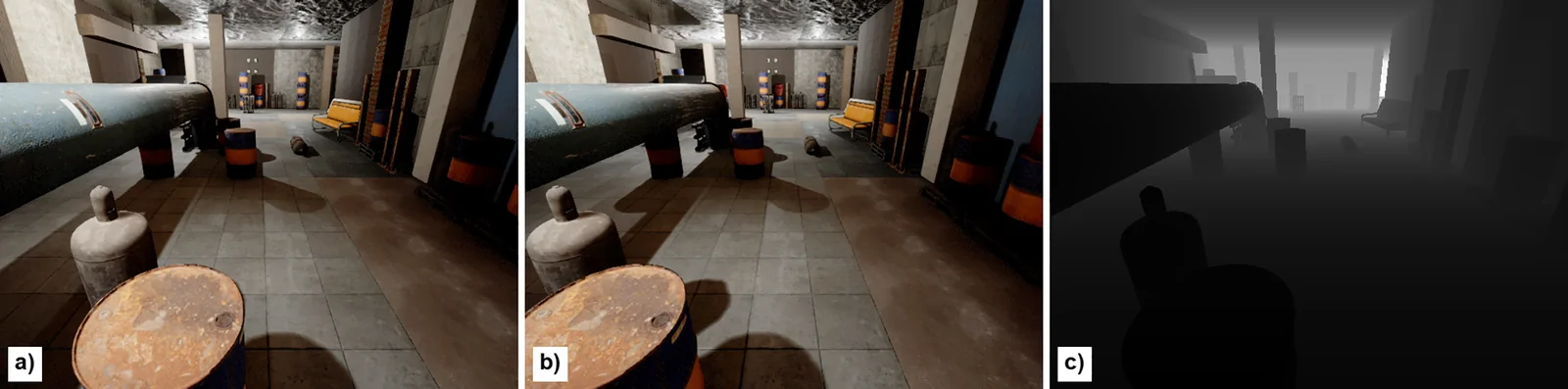
sequence06: direction 1, displaced, baseline 32 cm, parallel (552).

**Fig. 11:**
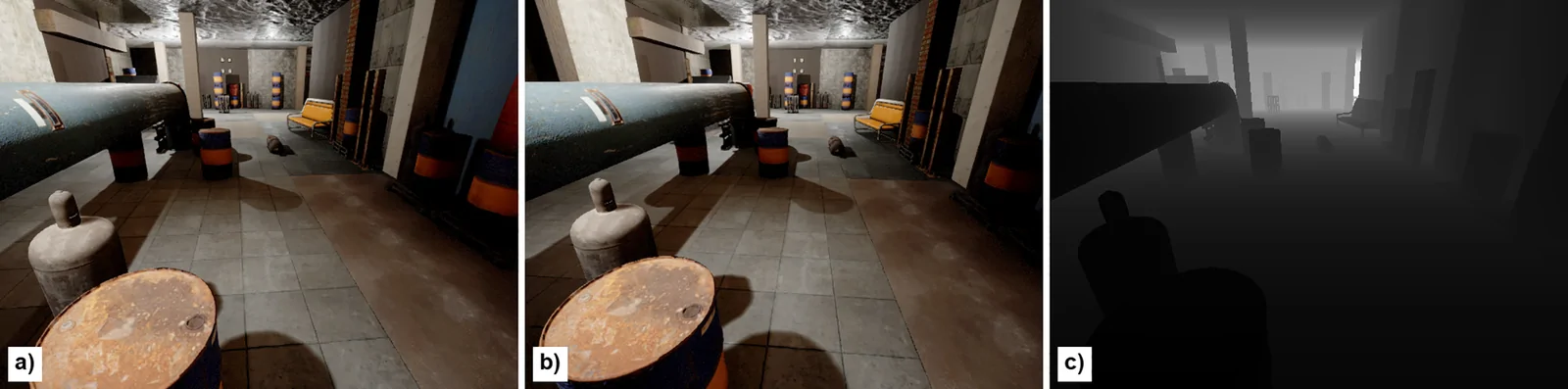
sequence07: direction 1, centred, baseline 24 cm, convergent cameras (5° each) (546).

**Fig. 12:**
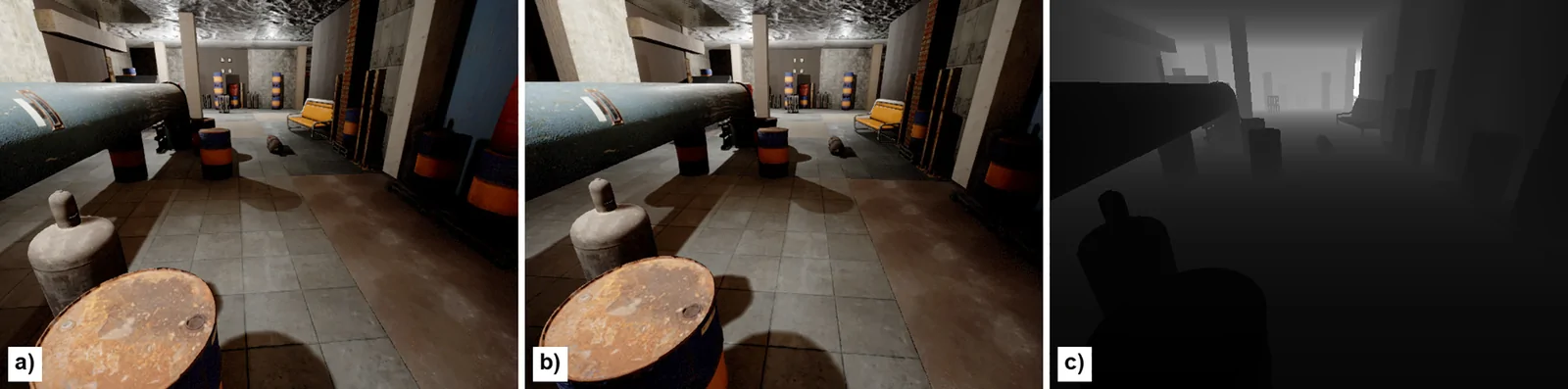
sequence08: direction 1, displaced, baseline 24 cm, convergent (5° each) (566).

Scene 2 (8 sequences, total 5623 stereo pairs). The corresponding samples for Scene 2 are shown in Fig. 13, Fig. 14, Fig. 15, Fig. 16, Fig. 17, Fig. 18, Fig. 19, Fig. 20.

**Fig. 13:**
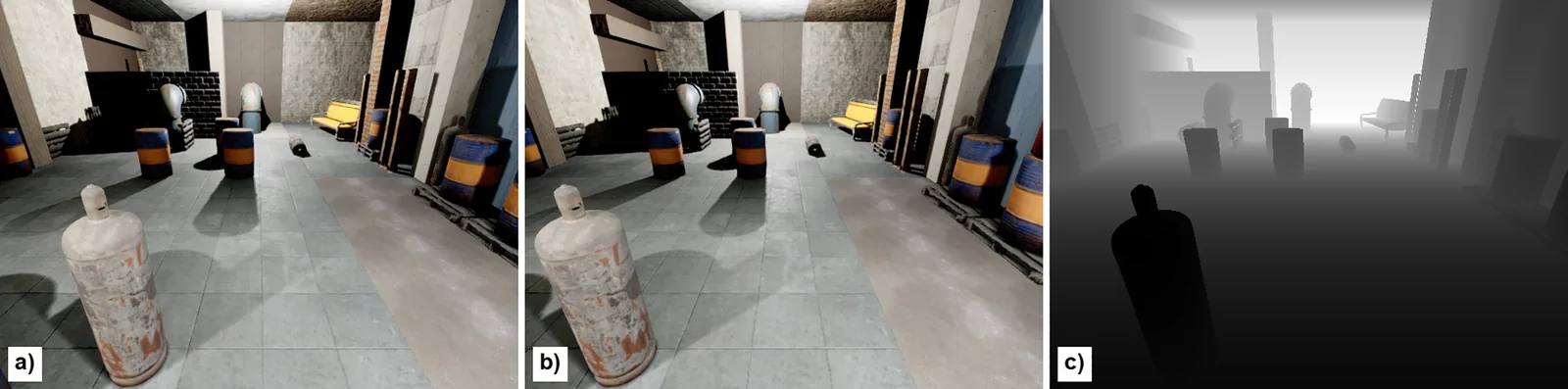
sequence01: direction 1, centred, baseline 24 cm, parallel cameras (709).

**Fig. 14:**
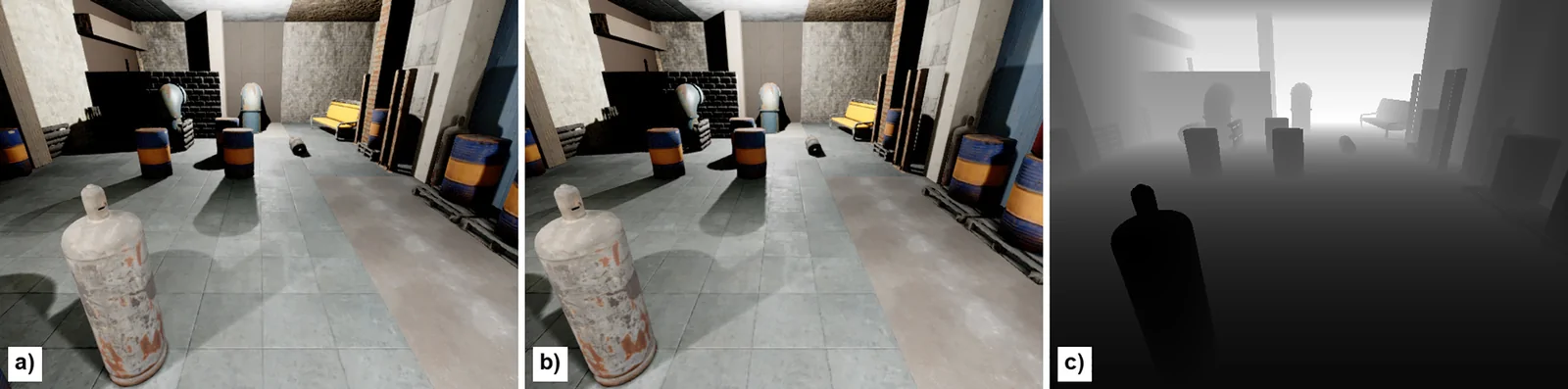
sequence02: direction 1, displaced, baseline 24 cm, parallel (699).

**Fig. 15:**
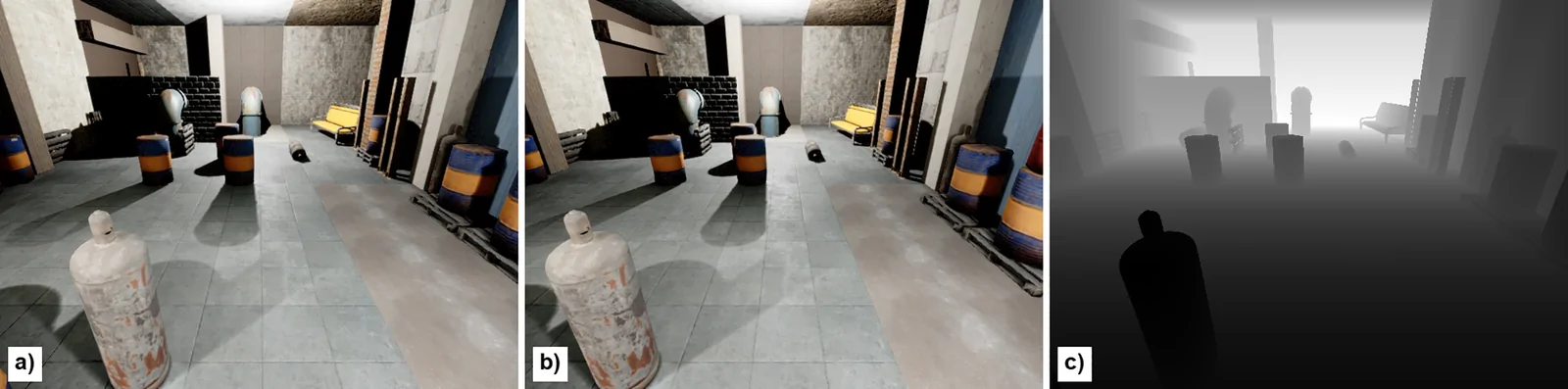
sequence03: direction 2, centred, baseline 24 cm, parallel (706).

**Fig. 16:**
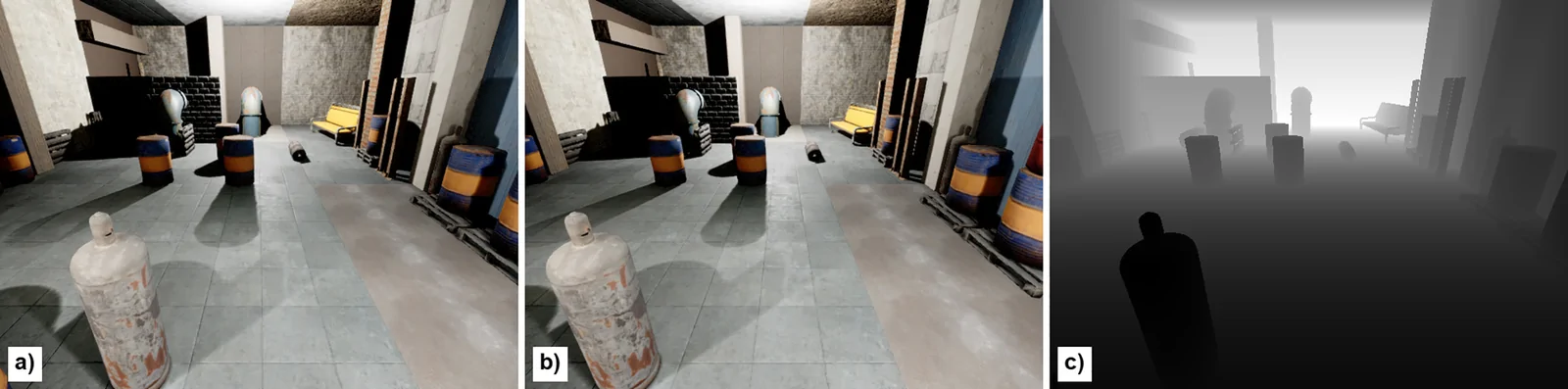
sequence04: direction 2, displaced, baseline 24 cm, parallel (721).

**Fig. 17:**
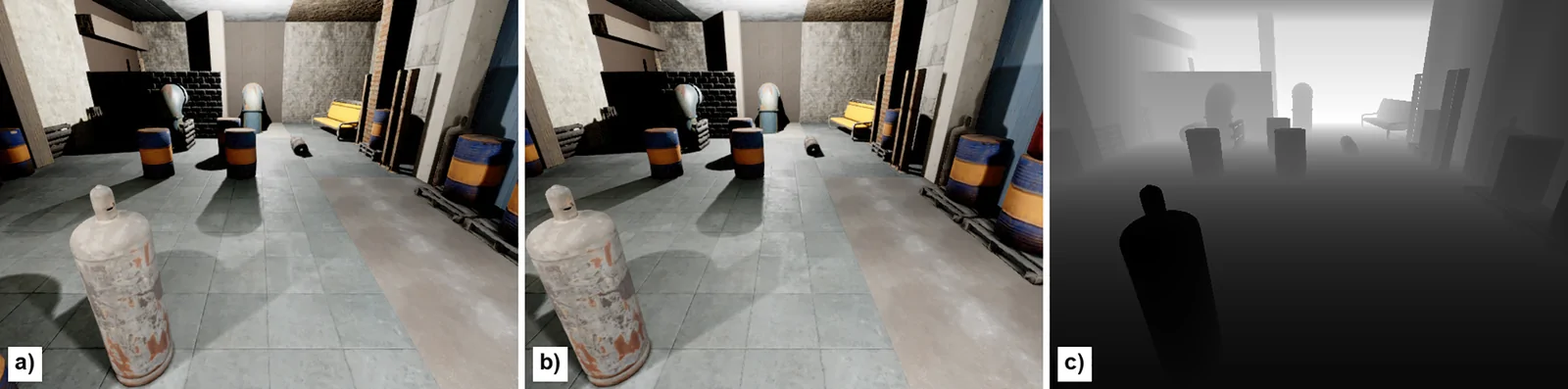
sequence05: direction 1, centred, baseline 32 cm, parallel (692).

**Fig. 18:**
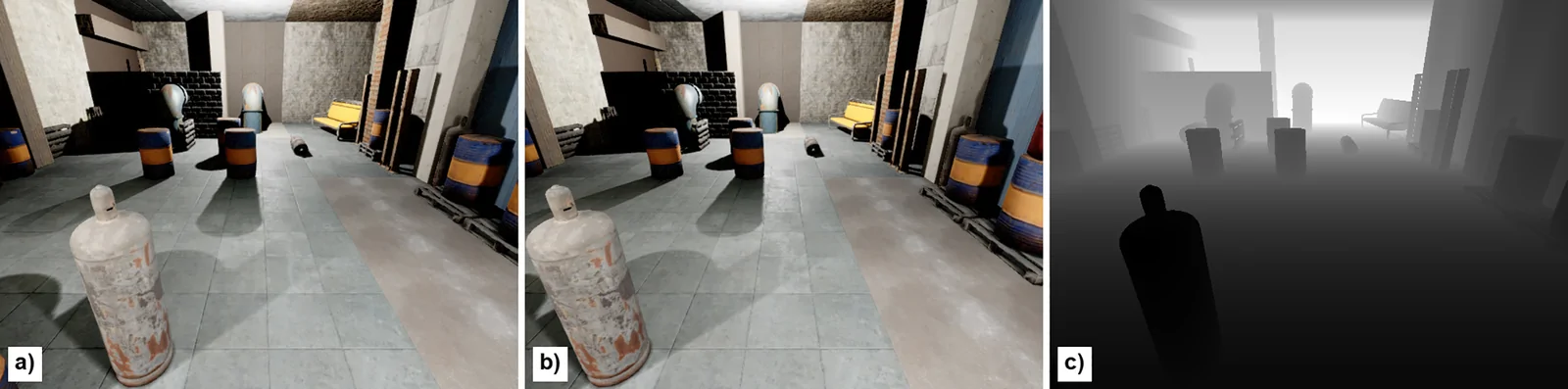
sequence06: direction 1, displaced, baseline 32 cm, parallel (710).

**Fig. 19:**
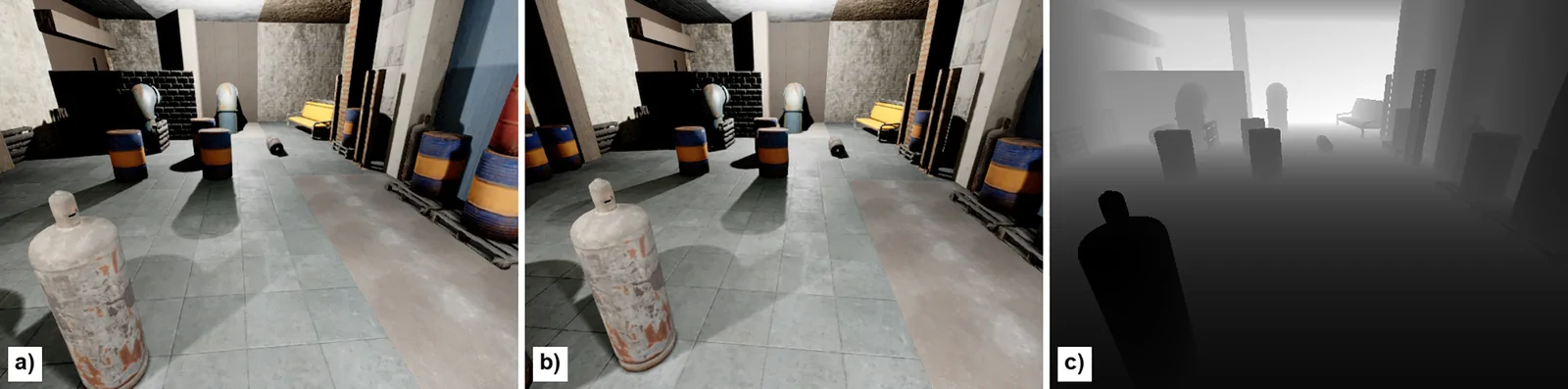
sequence07: direction 1, centred, baseline 24 cm, convergent cameras (5° each) (692).

**Fig. 20:**
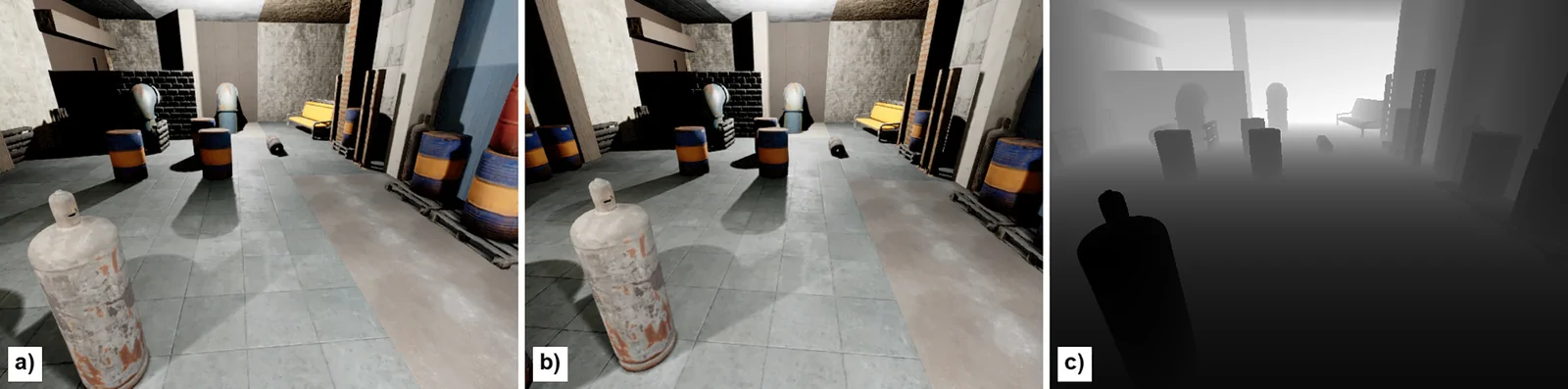
sequence08: direction 1, displaced, baseline 24 cm, convergent (5° each) (694).

Scene 3 and Scene 4 follow the same route configurations adopted in Scene 1 and Scene 2, respectively, while using the continuous-velocity acquisition procedure described in this section, which produces smoother trajectory transitions, together with denser temporal sampling and slight environmental variations. Both scenes preserve the same controlled variations in loop direction, lateral drone displacement, stereo baseline, and camera convergence.

Scene 3 contains eight acquisition sequences and 12,317 synchronized stereo pairs. It follows the same route configuration adopted in Scene 1, including centered and laterally displaced trajectories, 24 cm and 32 cm stereo baselines, parallel stereo cameras, and convergent stereo cameras with 5° inward rotation each.

Scene 4 contains eight acquisition sequences and 14,491 synchronized stereo pairs. It follows the same route configuration adopted in Scene 2, preserving the same acquisition variations while providing smoother curve transitions and denser temporal sampling.

The increased temporal density and smoother trajectory transitions in Scene 3 and Scene 4 support more detailed evaluation of temporal consistency, stereo correspondence stability, and trajectory robustness in visual odometry and SLAM pipelines.

## 4 Experimental Design, Materials and Methods

Four indoor scenes were created in Unreal Engine 4.27 using realistic materials, lighting, and structural elements. A stereo camera rig was mounted on a virtual drone within the AirSim simulation framework. Camera intrinsics were fixed for all sequences, while stereo baseline and camera convergence were varied according to the acquisition configuration. The simulation and rendering configuration is summarised in Table 4. Data acquisition was performed at approximately 3 Hz for Scenes 1 and 2 and at approximately 10 Hz for Scenes 3 and 4. The drone followed predefined closed-loop trajectories under controlled motion conditions. Complete timestamped 6-DoF poses are provided for every sequence, allowing the realized motion and velocity profile to be reconstructed directly from the ground-truth trajectory data.

Each sequence follows a scripted closed-loop trajectory, in which the drone takes off, traverses the predefined path, and lands at the same initial location. Python scripts control the drone motion and record synchronized left and right RGB images, depth maps, and camera poses via the AirSim API. All timestamps correspond to the left camera, which serves as the reference stream for stereo correspondence and pose information.

Two acquisition procedures were used. In Scenes 1 and 2, the vehicle is commanded through successive position and heading targets: it flies to each waypoint, stops, rotates in place towards the next heading, and then proceeds. Smooth heading transitions are obtained by densifying the waypoint list, so that each turn is decomposed into a sequence of small heading increments. In Scenes 3 and 4, the vehicle is instead driven by continuous velocity commands, with the heading interpolated along each segment and constrained by a commanded maximum yaw rate of 18°/s, which removes the intermediate stops. In both procedures the capture loop runs independently of the motion controller, and every frame carries a single timestamp shared by the left image, the right image, the depth array and the pose. Scenes 3 and 4 therefore provide smoother inter-frame motion, while Scenes 1 and 2 contain pure-rotation segments useful for evaluating robustness to rotational motion.

The scenes are static and the acquisition involves no stochastic component, so trajectories are geometrically repeatable across runs. Frame capture is paced against wall-clock time, which accounts for the small differences in frame count between otherwise equivalent sequences reported in Table 2.

**Table 2:** Complete list of the 32 acquisition sequences, with loop direction, rig placement, stereo baseline, camera convergence, sampling rate and number of stereo pairs. Displaced routes are laterally offset with respect to the centred ones.

Scene	Sequence	Dir.	Placement	Baseline (cm)	Convergence	Rate (Hz)	Pairs
1	sequence01	1	Centred	24	Parallel	3	532
1	sequence02	1	Displaced	24	Parallel	3	522
1	sequence03	2	Centred	24	Parallel	3	548
1	sequence04	2	Displaced	24	Parallel	3	581
1	sequence05	1	Centred	32	Parallel	3	560
1	sequence06	1	Displaced	32	Parallel	3	552
1	sequence07	1	Centred	24	Convergent (5° each)	3	546
1	sequence08	1	Displaced	24	Convergent (5° each)	3	566
	Scene 1 total						4407
2	sequence01	1	Centred	24	Parallel	3	709
2	sequence02	1	Displaced	24	Parallel	3	699
2	sequence03	2	Centred	24	Parallel	3	706
2	sequence04	2	Displaced	24	Parallel	3	721
2	sequence05	1	Centred	32	Parallel	3	692
2	sequence06	1	Displaced	32	Parallel	3	710
2	sequence07	1	Centred	24	Convergent (5° each)	3	692
2	sequence08	1	Displaced	24	Convergent (5° each)	3	694
	Scene 2 total						5623
3	sequence01	1	Centred	24	Parallel	10	1511
3	sequence02	1	Displaced	24	Parallel	10	1563
3	sequence03	2	Centred	24	Parallel	10	1572
3	sequence04	2	Displaced	24	Parallel	10	1575
3	sequence05	1	Centred	32	Parallel	10	1497
3	sequence06	1	Displaced	32	Parallel	10	1549
3	sequence07	1	Centred	24	Convergent (5° each)	10	1494
3	sequence08	1	Displaced	24	Convergent (5° each)	10	1556
	Scene 3 total						12,317
4	sequence01	1	Centred	24	Parallel	10	1804
4	sequence02	1	Displaced	24	Parallel	10	1777
4	sequence03	2	Centred	24	Parallel	10	1909
4	sequence04	2	Displaced	24	Parallel	10	1844
4	sequence05	1	Centred	32	Parallel	10	1804
4	sequence06	1	Displaced	32	Parallel	10	1757
4	sequence07	1	Centred	24	Convergent (5° each)	10	1810
4	sequence08	1	Displaced	24	Convergent (5° each)	10	1786
	Scene 4 total						14,491
	All scenes						36,838

The Python scripts implementing both acquisition procedures, together with the AirSim configuration file, are provided in the dataset repository.

Per-sequence association files (associate_rgb.txt and associate_rgbd.txt) were generated to guarantee one-to-one correspondence between stereo image pairs and between RGB images and depth data. Ground-truth trajectories are provided in quaternion form in groundtruth.txt. Together with the extrinsic parameters listed in Table 3, they enable direct reuse of the dataset in visual SLAM and odometry evaluation pipelines, in either the body frame or the camera optical frame.

**Table 3:** Intrinsic and extrinsic parameters of the virtual stereo rig. Intrinsics are exact by construction and identical for both cameras. Translations are given in the vehicle body frame, in which the Z axis points downwards.

Parameter	Value
Image resolution	640×480 px
Horizontal field of view	90.0°
fx, fy	320.0 px
cx, cy	320.0 px, 240.0 px
Lens distortion	None; ideal pinhole projection (k1 = k2 = k3 = p1 = p2 = 0)
Stereo baseline B	0.24 m (sequences 01–04, 07–08); 0.32 m (sequences 05–06)
fx B	76.8 m px (B = 0.24 m); 102.4 m px (B = 0.32 m)
Left camera translation	X + 1.00 m, Y −0.12 m, Z + 0.10 m (body frame, NED)
Right camera translation	X + 1.00 m, Y + 0.12 m, Z + 0.10 m (body frame, NED)
Camera rotation (parallel)	pitch −23.0°, roll 0.0°, yaw 0.0°
Camera rotation (convergent)	pitch −23.0°, roll 0.0°, yaw −5.0° / +5.0°
Depth image type	DepthPlanar, 32-bit float, metres, left camera

**Table 4:** Unreal Engine and AirSim configuration used to generate the dataset.

Item	Configuration
Game engine	Unreal Engine 4.27
Simulation framework	Microsoft AirSim (Multirotor, SimpleFlight)
Clock speed	1.0 (real time)
World scale	100 Unreal units per metre
Scene assets	AirSim Content, Unreal Starter Content, Quixel Megascans (2K/4 K)
Static lighting	39 lightmaps; packed light and shadow map resolution 1024
Local lighting	19 static point lights with per-actor intensity and attenuation radius
Sky and atmosphere	Sky Sphere and Atmospheric Fog
Reflections	Sphere Reflection Capture, influence radius 3000, brightness 1.0
Post-processing	Engine default settings; no post-process volume placed in the level
Hardware	Intel Core i7–10750H laptop, 32 GB RAM, NVIDIA RTX 2070 Max-Q
Acquisition time	Approximately 3 min per sequence

No radiometric or geometric preprocessing is applied to the RGB images: frames are retrieved uncompressed from the simulator as 8-bit three-channel arrays and written directly to PNG. The only operation applied to the depth arrays is the replacement of non-finite values by zero, which prevents downstream failures without altering valid measurements.

## Limitations

Although the dataset includes multiple trajectory configurations and acquisition conditions, it is restricted to indoor synthetic environments, and the images inherit the properties of rendered imagery. They contain no sensor noise, rolling-shutter effects, or exposure-related motion blur, and the virtual cameras follow an ideal pinhole projection without lens distortion or vignetting, so geometry is exact by construction rather than by calibration. The depth maps are correspondingly dense and exact throughout the field of view.

The environments are static, with baked lighting and no moving objects, people, or illumination changes over time; weather effects are not applicable to the indoor setting.

These properties are deliberate. By removing the sources of variability that confound real acquisitions, the dataset isolates the effect of stereo geometry and trajectory conditions, which is precisely what its controlled acquisition configurations are designed to exploit. Results obtained here characterise algorithm behaviour under exact geometry, and are complementary to evaluation on real imagery, where sensor and environmental effects come into play.

## Ethics Statement

The authors confirm that the dataset complies with all ethical requirements for publication in Data in Brief. This work does not involve human subjects, animal experiments, or data collected from social media platforms.

## Credit Author Statement

Yan Medeiros Morona: Conceptualization; Methodology; Software; Data curation; Visualization; Writing – original draft.

Tiago Loureiro Figaro da Costa Pinto: Supervision; Methodology; Writing – review and editing.

Daniel Juchem Regner: Visualization; Writing – review and editing.

## Data Availability

Hugging FaceUE4-Stereo (Original data)
